# Supplementary material for: A mixed methods evaluation of a pilot open trial of a mentor-guided digital intervention for youth anxiety
Source: PLOS Digit Health. 2026 Feb 23;5(2):e0001187. doi: 10.1371/journal.pdig.0001187 (PMC12928454; doi:10.1371/journal.pdig.0001187)
Supplement: S1 Text — 1: Methods Notes: 1: Additional Recruitment Information, 2: Mentor Training Details, 3: Additional CBM-I Training Tasks. 2: Results Notes: 1: Mentor/Mentee Relationship Quality, 2: Deviations from Pre-registration. 3: Interview Guides: 1: Mentee Interview Guide, 2: Mentor Interview Guide. 4: Supplementary Tables: S1 Table A: Data Triangulation Table, S1 Table B: Measure Administration Schedule. 5: Supplementary Figures: S1 Fig A: Initial Thematic Map: Mentee Version, S1 Fig B: Initial Thematic Map: Mentor Version. (DOCX) [file pdig.0001187.s001.docx]

**Supplemental Materials (S1 Text)**

**Table of Contents**

***1: Methods Notes***

1: Additional Recruitment Information

2: Mentor Training Details

3: Additional CBM-I Training Tasks

***2: Results Notes***

1: Mentor/Mentee Relationship Quality

2: Deviations from Pre-registration

***3: Interview Guides***

1: Mentee Interview Guide

2: Mentor Interview Guide

***4: Supplementary Tables***

S1 Table A: Data Triangulation Table

S1 Table B: Measure Administration Schedule

***5: Supplementary Figures***

S1 Fig A: Initial Thematic Map: Mentee Version

S1 Fig B: Initial Thematic Map: Mentor Version

**Section 1: Method Notes**

**1.1 Additional Recruitment Information**

Youth without a mentor were unable to participate, given the purpose of this study was to examine whether the proposed model (of training an existing mentor to provide supportive accountability to youth participating in a technology-delivered intervention) was acceptable and feasible to mentors and mentees. The recruitment process could be initiated by mentee, mentor, or parent. Regardless of which individual contacted the study team, interest was confirmed and the relevant contact information of all three consenting or assenting parties (parent, mentee, and mentor) was collected.

**1.2: Mentor Training Details**

During the mentor training, mentors were given a brief overview about youth anxiety and CBM-I, and specifically how CBM-I works to improve symptoms of youth anxiety. They were familiarized with the study background, procedures, and rationale, and instructed how to explain MindTrails-Teen to their mentees. Finally, they were provided with training in supportive accountability^1^a process-based strategy by which mentors are instructed to motivate their mentees through encouragement, support, and the defining of clear, individual goals with the intention of increasing intrinsic motivation and thus adherence to the intervention. Mentors were given access to a resource website throughout the intervention so that they might follow our suggested timeline and protocol for providing support.

Once mentor training was completed, the mentor was provided a copy of the training slides and instructions for explaining and downloading MindTrails Teen with their mentee. Initially, we had intended for mentors to download MindTrails Teen in-person with their mentees. While this occurred in some cases, many of our mentors did not meet regularly in person with their mentees, and therefore halfway through the study we began sending instructions and app download codes directly to mentees. In this case, mentors were asked to discuss MindTrails-Teen and confirm their mentee had downloaded the app at the next meeting or check-in. During these check-ins, mentors were asked to encourage their mentees to complete trainings and use skills learned in the app to reinforce patterns of flexible thinking. During in-person meetings, mentors were encouraged to discuss what the mentee had learned in the MindTrails Teen app, and how the mentee might apply some of their new flexible thinking skills to situations in their own lives.

**1.3: Additional CBM-I Protocol Tasks**

*Predict the Future Task:* The Predict the Future Task was administered immediately prior to the Lemon Exercise in Week 1, Session 1. In this task, mentees completed a guided visual imagery exercise asking them to imagine an upcoming anxiety-inducing scenario as vividly as possible. They were asked to write one or two sentences describing the situation, and then imagine the situation playing out as vividly as possible for 20 seconds. Finally, they were asked to take 60 seconds to think of as many different thoughts as possible, both positive and negative, that they might have while being in this specific anxiety-inducing situation. This task was intended to give mentees practice with flexible thinking and remind them of the possible positive interpretations of events. It was also available as a “Practice Task” for mentees to engage in during Week 1, 3, and 5 once they had completed their short training exercise.

*Remember the Past Task:* The Remember the Past task was available as a “Practice Task” for mentees during Week 2 and 4 once they had completed their long training exercise. In this task, they were asked to write about an anxiety-inducing event that had recently happened. They were asked to write one or two sentences describing the situation, and then imagine the situation playing out as vividly as possible for 20 seconds. Finally, they were asked to take 60 seconds to think of as many different thoughts as possible, both positive and negative, that they might have had or did have while being in this specific anxiety-inducing situation. This task was intended to give mentees practice with flexible thinking and remind them of the possible positive interpretations of events.

*Use Your Imagination Task:* The Use Your Imagination Task was administered immediately prior to training following all sessions after Session 1. This slightly shortened version of the Predict the Future task was intended to give mentees more practice in vividly imagining situations before starting training. Once they had something in mind, mentees were asked to write down the event in a few words, then to imagine the situation playing out as vividly as possible for 20 seconds. Following this, they were asked how anxious they felt (at the highest level of their anxiety) completing the task. This was measured with a sliding scale from 0-10, with 0 being absolutely no anxiety and 10 being totally anxious.

*Quick Thinking Task*: The Quick Thinking Task appeared as a Practice Task following completion of the mentees’ first half of weekly training (short CBM-I training task). The Quick Thinking tasks were designed to be very short, lasting no more than 2 minutes. During this task, mentees were shown an example of a text message that might be anxiety provoking (for example, a text message from a parent saying “You need to come home right now”). Following this image, mentees were asked to write down the different thoughts that might come to their mind in the moment while viewing the image. On the next screen, mentees were given one of “Quick Thinking” prompts, which showed a brief scenario and accompanying pictures (e.g. “Your teacher is about to hand back graded tests from last week, and you remember how hard the test was). Mentees were then asked to imagine themselves in this scenario and write down the many different thoughts that might come to mind. This was another method of practicing flexible thinking. In Session 1, this was the Abbreviated Lemon Exercise, a 5-minute guided imagery task intended to enhance imagined sensory experience. This task was intended to give mentees practice with immersing themselves in their imagined experience, a key mechanism of CBM-I.^2^

**Section 2: Results Notes**

**2.1: Mentor/Mentee Relationship Quality**

We collected data on mentor/mentee relationship quality at each time point using the Strength of Relationship Scale (SoR for mentors, Y-SoR for youth).^3^ The SoR and YSoR (14 and 10-item measures, respectively) are mentor and youth assessments of relationship quality, with positively and negatively valanced statements about the mentor/mentee relationship rated on a 5-point Likert scale from 1 *(not at all true)* to 5 *(always true).* Exploratory post-hoc analyses of the SoR and YSOR responses from pre-to-post intervention using cohen’s *d* with hedge’s *g* correction indicate a small to medium improvement in strength of relationship in both the PP (*n=*8) and ITT (*n*=14) samples (PP: Hedge’s *g*=-0.64, 95% CI:[-1.68, 0.40]; ITT: Hedge’s *g*=-0.48, 95% CI:[-1.28, 0.32].

**2.2: Deviations From Preregistrations**

We report several minor deviations from our qualitative (https://osf.io/nvukg/) and quantitative (http://osf.io/bqc2e/) preregistrations. Regarding the qualitative preregistration, we report additions from our proposed analysis plan. The original analysis plan presented in the pre-registration was followed with fidelity, but following further research into best practices for thematic analysis the team decided to conduct the follow-up step of thematic mapping (detailed in the main manuscript). This process reduced the size of our codebook as it involved condensing some codes and expanding others into new themes. See the main manuscript for more information on this process. Our other deviation from the preregistration is that we did not engage in one of our proposed credibility strategies, member checking, as we were unable to get in touch with those participants we were interested in interviewing.

Regarding the quantitative pre-registration, we conducted several post-hoc analyses not included in the pre-registration. For our primary analysis of intervention outcome, we did not calculate a trend line on our data visualizations as we determined our sample size was too small for trends to be meaningful. In order to better characterize our sample, we examined measures of central tendency (mean, mean, and mode) for number of sessions completed. In addition, we performed exploratory analyses of the SoR and YSoR responses from pre-to-post intervention using cohen’s *d* with hedge’s *g* correction. These analyses were conducted to examine change in mentor/mentee relationship from both mentor and mentee perspective across the study. These analyses were not pre-registered as they were not related to a primary hypothesis.

**Section 3: Interview Guide**

**3.1: Mentee Interview Guide**

**QUESTIONS**

**App Appearance/Functionality/Usability**

1. Tell us about your experience using the MindTrails Teen app.
   1. What was your favorite part of the app?
   2. What was your least favorite part of the app?
2. Describe your thoughts about how the app looked.
   1. Is there anything you would change about how the app looked?
3. Were there any parts of the app that were hard to use?
4. We want the app to be helpful for as many teens as possible. Sometimes, people tell us apps aren’t helpful because parts of the app don’t seem to fit their background or parts of their identity. What do you think about that? Were there any parts of the app that you felt weren’t a good fit for you based on aspects of your identity?
5. Would you recommend the MindTrails Teen app to other teenagers struggling with anxiety? Why or why not?
6. Overall, how would you describe the MindTrails Teen app to a friend?

**Engagement**

1. How often did you use the app?
   1. What were some things that made it hard to use the app?
   2. What are some things we could change about the app so that teens like you are more likely to use it?
2. How easy was it for you to pay attention when using the app? What could we change to make it (even) more interesting?

**Training Tasks and Domains**

1. What are your thoughts about the short training tasks you completed?
   1. As a reminder, these were tasks that asked you to choose a word that completed a brief story
   2. What did you like? What did you dislike? What suggestions do you have for improvement?
2. What are your thoughts about the long training tasks you completed?
   1. As a reminder, these were tasks that asked you to picture yourself in a situation and write down helpful thoughts, feelings, and behaviors
   2. What did you like? What did you dislike? What suggestions do you have for improvement?
3. Did you like the **short training tasks** or the **long** **training tasks** better?
4. In the program, we targeted anxiety around the following topics
   1. Academics
   2. Social Situations
   3. Social Media
   4. Home Life
   5. General (A loud sound, auditioning for a play, exploring a narrow cave)
      1. Did you enjoy these situations? Did you feel like they were things that someone your age would experience?
      2. Have you ever experienced stress tied to your identity?
      3. What do you think about having those experiences included in an app like this?

**Mentor Guidance/Relationship**

1. Do you feel like your mentor was helpful in encouraging you to use the app? If yes, how so? If no, why?
2. How did using the MindTrails Teen with your mentor change your relationship with them?
3. How often did you talk about MindTrails Teen with your mentor?

**3.2: Mentor Interview Guide**

**QUESTIONS**

**Mentee Experience**

1. What was it like incorporating MindTrails Teen app into your mentoring sessions?
2. What was challenging about incorporating the MindTrails Teen app into your mentoring sessions?
3. What was helpful about incorporating the MindTrails Teen app into your mentoring sessions.
4. How much do you think your mentee enjoyed the MindTrails app?
   1. What did they like about it?
   2. What did they dislike about it?

**Relationship with Mentee**

1. In what ways, if any, did participating in the MindTrails program change your relationship with your mentee?
2. How did using the MindTrails teen app impact your ability to have conversations with your mentee about their mental health?
3. How often did you talk about MindTrails Teen with your mentee?

**General Feedback**

1. What are additional resources that would be helpful for you as a mentor to guide your mentee in the MindTrails Teen program?
2. Did you feel like you had enough training to support your mentee in the MindTrails Teen program?
   1. [If Yes] What was helpful?
   2. [If No] What was unhelpful?
3. What other training or support would be helpful for you as a mentor?
4. Is there anything you would change about the MindTrails Teen program?
5. What did you like about the MindTrails Teen program?
6. What did you dislike about the MindTrails Teen program?

**Section 4: Supplementary Tables**

**S1 Table A.**

|  | Quantitative Data | Qualitative Data |
| --- | --- | --- |
| Intervention feasibility | - Protocol Adherence (Mentees)   - Percentage of enrolled mentees that complete at least ½ of assigned CBM-I training sessions   - Percentage of enrolled mentees the complete all assigned CBM-I sessions - Clinical deterioration (Mentees)   - Percentage of mentees who experienced an increase in symptoms on the anxiety subscale of the GAD-2 > 50% above their pre-intervention score | Mentee interview  “Were there any parts of the app that were hard to use?”  “How often did you use the app?” |
| Implementation framework feasibility | - Protocol Adherence (Mentors & Mentees)   - Percentage of mentor/mentee check-ins completed   - Number of times dyads discussed MindTrails during competed check-ins | Mentor Interview  “What was it like incorporating MindTrails Teen app into your mentoring sessions?”  “How often did you talk about MindTrails Teen with your mentee?”  Mentee Interview  “How often did you talk about MindTrails Teen with your mentor?” |
| Intervention acceptability | - Average responses to the following items on the MAQ: Mentee version   - “I enjoyed using the MindTrails App.”   - “I would recommend MindTrails to other mentees or teens.”   - “MindTrails changed the way I think during anxiety-provoking situations.” | Mentee Interview  “Tell us about your experience using the MindTrails Teen app. What was your (favorite/least favorite) part of the app?” |
| Implementation framework acceptability | - Average responses to the following items on the MAQ: Mentee version   - “I enjoyed working with my mentor to apply the skills I learned in MindTrails.”.”   - “I would recommend using MindTrails with a mentor to other mentees or teens.”   - “It was helpful to have my mentor support my use of MindTrails.” - Average responses to the following items on the MAQ: Mentor version   - “I enjoyed participating in this project.”   - “I felt prepared to help my mentee use MindTrails.”   - “I would recommend MindTrails with mentor assistance to other mentees.” | Mentor Interview  “What was (challenging/helpful) about incorporating the MindTrails Teen app into your mentoring sessions?”  “In what ways, if any, did participating in the MindTrails program change your relationship with your mentee?”  Mentee Interview  “Do you feel like your mentor was helpful in encouraging you to use the app? If yes, how so? If no, why?”  “How did using the MindTrails Teen with your mentor change your relationship with your mentor?” |
| Intervention outcomes | - Pre- to post-intervention change in target engagement (positive and negative interpretation bias) on the Recognition Rating Task - Pre- to post-intervention change in anxiety symptoms on the GAD-2. | Mentee Interview  “Would you recommend the MindTrails Teen app to other teenagers struggling with anxiety? Why?”  “Overall, how would you describe the MindTrails Teen app to a friend?” |

**S1 Table B**

Measure Administration Schedule

| **Measure** | **Eligibility Questionnaire (Mentee Only)** | **Baseline** | **Midpoint** | **Endpoint** | **1-month follow-up** | **Mentor Weekly Survey** |
| --- | --- | --- | --- | --- | --- | --- |
| Generalized Anxiety Disorder-7 (GAD-7) | Y |  |  |  |  |  |
| Self-Reported Anxiety Diagnosis | Y |  |  |  |  |  |
| Demographics | Y | X |  |  |  |  |
| The Patient Health Questionnaire-4 (PHQ-4) |  | X/Y | X/Y | X/Y | Y |  |
| Strength of Relationship Scale (SoR) |  | X | X | X |  |  |
| Youth Strength of Relationship Scale (YSoR) |  | Y | Y | Y | Y |  |
| Recognition Rating Task |  | Y | Y | Y | Y |  |
| MindTrails Acceptability Questionnaire (MAQ) |  |  |  | X/Y |  |  |
| Mentor Weekly Survey |  |  |  |  |  | X |

*Note: X indicates measure administered to mentor, Y indicates measure administered to mentee*

S1 Fig A: Initial Thematic Map: Mentee Version


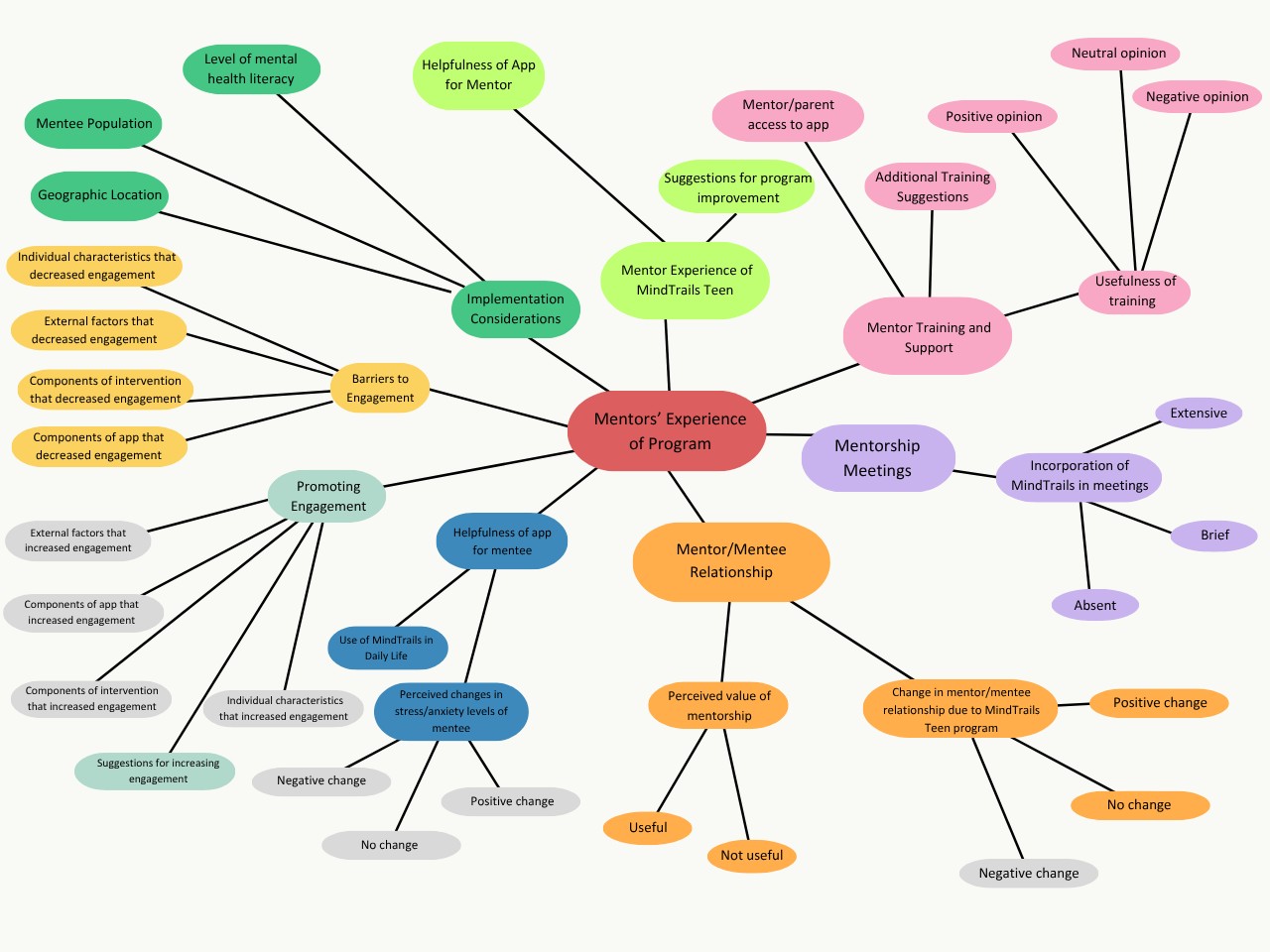


S1 Fig B: Initial Thematic Map: Mentor Version


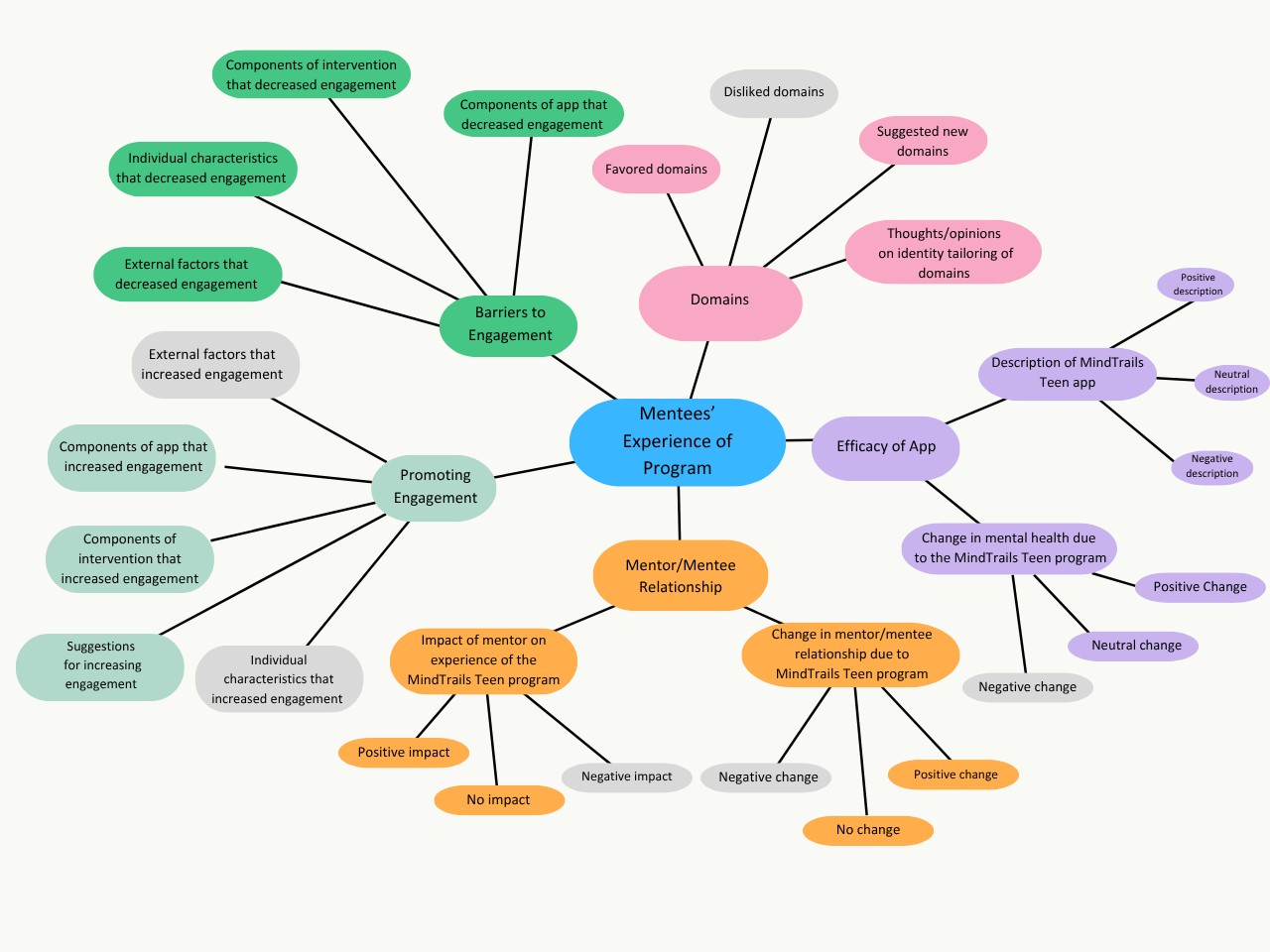


**References**

1. Mohr DC, Cuijpers P, Lehman K. Supportive accountability: a model for providing human support to enhance adherence to eHealth interventions. J Med Internet Res. 2011 Mar 10;13(1):e30.

2. Beard C. Cognitive bias modification for anxiety: current evidence and future directions. Expert Rev Neurother. 2011 Feb;11(2):299–311.

3. Rhodes JE, Schwartz SEO, Willis MM, Wu MB. Validating a Mentoring Relationship Quality Scale: Does Match Strength Predict Match Length? Youth & Society. 2017 May 1;49(4):415–37.
