# Supplementary material for: A mixed methods evaluation of a pilot open trial of a mentor-guided digital intervention for youth anxiety
Source: PLOS Digit Health. 2026 Feb 23;5(2):e0001187. doi: 10.1371/journal.pdig.0001187 (PMC12928454; doi:10.1371/journal.pdig.0001187)
Supplement: Attachment — Submitted filename: Response to Reviewers.docx [file pdig.0001187.s003.docx]

Response to Reviewers

We thank the reviews for their insightful comments. Please see below for our response to the comments and proposed changes:

1) “I recommend clarifying the rationale behind selecting the 13 to 17 age group. According to the United Nations, there is no universally agreed-upon definition of "youth." However, for statistical purposes, the UN defines youth as individuals aged 15 to 24 years. It would be helpful to explain how your chosen age range aligns with or diverges from this definition.”
   a. We thank the reviewers for this important point. Though we apply the term “youth” throughout the manuscript, this study was specifically focused on adolescents in their teenage years. We selected this specific age group (13-17) to align with the developmental norms of teenagers in United States, from where our sample was recruited. U.S. minors are legally defined as individuals below the age of 18, and most U.S. teenagers enter high school (e.g., 8^th^-12^th^ grades) between the ages of 13 and 14. Given the dynamic nature of anxiety symptoms and triggers across different stages of youth development (e.g., likelihood that romantic partners and social media would be a source of anxiety), we chose to tailor the content of the app to be appropriate for adolescents in this developmental stage. These clarifying details have been added to the manuscript (Page 9, lines 209-212).
2) “Please ensure that data is included in rows 217–220 of the table.”
   a. We are unsure to which table the reviewer is referring but have double-checked all tables to ensure necessary data is included and no data was mistakenly omitted.
3) “The sampling approach uses a convenience sampling resulting to a very low sample size, explain any strategy or strategies put in place to enhance representativeness, reduce bias and broaden the reach.”
   a. We have expanded the section characterizing our recruitment process and associated barriers (see Page 8-9; lines 208-215).
4) “In line 262, it was mentioned that all participants completed the demographic questionnaire, this contradicts what was presented earlier in the study as well as in table 1.”
   a. We thank the reviewer for noting this inconsistency, and have clarified the wording on Page 12, Line 266.
5) “Could you clarify the specific stage at which the reduction in anxiety symptoms was measured? was this outcome assessed at the conclusion of the intervention or during a subsequent follow-up period?”
   a. This has been clarified in the manuscript on page 25, lines 448-451.

We would also like to note that we have updated the colors of three figures (Fig_1.tif, Fig_2.tif, Fig_3.tif) to increase clarity and added a note regarding interpretation. Once again, thank you for your time and thoughtful review of the manuscript. We look forward to hearing from you.

Sincerely,

Emma Wolfe, M.A.
