# Supplementary material for: A mixed methods evaluation of a pilot open trial of a mentor-guided digital intervention for youth anxiety
Source: PLOS Digit Health. 2026 Feb 23;5(2):e0001187. doi: 10.1371/journal.pdig.0001187 (PMC12928454; doi:10.1371/journal.pdig.0001187)
Supplement: Attachment — Submitted filename: Response to Reviewers_2.docx [file pdig.0001187.s004.docx]

**Response to Reviewers**

**Reviewer 3**

1. The small sample size is acknowledged as a pilot limitation, but more discussion is needed on how this affects generalizability, especially given the racial homogeneity and limited geographical spread of participants.

We agree and have updated the limitations section to better reflect the limitations of the small and racially homogenous sample (pg. 47-48).

“Although the study had multiple strengths, including its mixed methods design, recruitment of an anxious sample, and innovative implementation framework, there were also several limitations. First, our sample of mentors was racially and ethnically homogenous (primarily White and non-Hispanic/Latino) and, though our sample of mentees had slightly more racial and ethnic diversity than the mentors, it was still not fully representative of the national mentee population. Furthermore, while our sample had unique representation from underserved rural communities (Iowa, Nebraska, South Texas) it did not contain broad geographic diversity. Second, our small sample size significantly limits the generalizability of our findings. More research is needed with large, geographically and demographically diverse samples randomized to MindTrails Teen vs. a comparison condition before making conclusive recommendations about the efficacy of the intervention and the feasibility of the implementation pathway.”

2. The rationale for using mentor-guided delivery of a DMHI is compelling and timely; however, clarify whether mentors had any prior training or experience in delivering psychological interventions beyond the brief training provided.

This question was not specifically posed to mentors, but no experience of this kind was reported to our study team during mentor training or follow-up qualitative interviews. We have included this clarification in the manuscript (pg. 12). Notably, the fact that this training is routinely not a part of mentor programs was a motivation for the study as some mentors express concern that they are not well prepared to support mentees who are struggling.

“It is unknown whether mentors had any training or previous experience delivering psychological interventions, but none was reported to the study team during the pilot trial.”

3. Consider expanding on why negative interpretation bias did not change—was this likely due to the psychometric flaws in the task or limitations in the intervention content?

We have provided additional detail to our explanation of this finding in the manuscript (pg. 46). See below:

“Given the relative consistency of interpretation bias change observed in prior trials of digital CBM-I, we suspect that the poor internal consistency of our novel measure of interpretation bias is likely to explain our mixed findings for bias change. We adapted the Recognition Rating Task (our measure of interpretation bias, which was originally designed for adults) so the scenario content was more suitable for adolescents. Our post-hoc discovery of the measure’s poor internal consistency highlights the importance of selecting psychometrically valid instruments.”

4. Table 2 is well-structured, but there are readability issues in formatting. Consider separating qualitative and quantitative benchmarks to improve clarity.

We thank the reviewer for this feedback. We have separated Table 2 into three separate tables (Table 2: Feasibility Benchmarks, Table 3: Acceptability Benchmarks, Table 4: Intervention Outcome Benchmarks) to improve clarity.

5. The selection of the 13–17 age group is now explained, but referencing specific developmental literature on adolescent cognitive-emotional development would strengthen the justification.

We thank the reviewer for this suggestion. Additional justification has been provided based on a brief review of the adolescent cognitive-emotional development literature on pg. 9:

“During this stage of development, most adolescents will experience key cognitive-emotional transitions, including a desire for autonomy and self-reliance and the ability to engage in complex forms of metacognition (Way-Lake, Crouter, & McHale, 2010; Weil et al., 2013). The latter can lead to an increase in self-focused rumination and is associated with the development of anxiety disorders in older adolescents (Wells & Carter, 1999).

6. The manuscript would benefit from a short paragraph on implications for scalability—especially since mentor-based models may limit reach in real-world settings.

We have updated the manuscript (pg. 50) with more discussion of scalability implications. See below:

“Finally, in addition to refining the mentorship supportive accountability model, future research should explore other implementation pathways and potential options for scaling the intervention. While the mentorship implementation framework holds promise for increasing supportive accountability and adherence, and may increase access by providing a point of care in spaces where teens already interact, scaling of the mentorship implementation framework is inherently limited by mentor availability and youth having access to participating mentorship programs.

7. Include limitations related to potential social desirability bias in mentor and youth reporting, especially as dyads had established relationships prior to study participation.

We thank the reviewer for identifying this important limitation. We have updated the manuscript to address this concern (pg. 47).

“Third, it is possible that social desirability bias and demand effects may have accounted for some of our findings, with teens feeling some pressure to report positive changes in symptoms and their mentor-mentee relationship following the program, especially given the pre-established relationships between mentors and mentees. Future work should measure the influence of social desirability on the intervention effects, and take additional steps to reduce demand effects, such as having an independent assessor.”

8. Given the pilot design, consider framing the positive findings on anxiety reduction more cautiously—refrain from implying causality without a control group.

We have softened this language throughout the manuscript.

Reviewer #4:

This manuscript describes a very small, single-group, pre/post pilot study of a cognitive bias app for anxiety among teens receiving mentorship. Overall, while the study could make a modest contribution to the literature, a few major concerns should be addressed.

1.) The weakness of the cognitive bias measure remains understated. Although the manuscript repeatedly says that the relevant findings "should be interpreted with caution," a reliability of a=0.35 suggests that the measure largely reflects error and likely cannot be interpreted at all. If reliability was really this low, then it would seem any findings (e.g., pre-post effect sizes) are mostly random. If the authors elect to retain these analyses, they should thoroughly and meaningfully address what any findings based on it mean, given that the measure is largely error.

We thank the reviewer for this point and have softened our language interpreting this finding throughout the discussion.

2.) Reliabilities for other measures should be reported as well, especially the PHQ-9 anxiety questions.

We have updated the measures section to include reliability scores from the literature for the PHQ-4 anxiety subscale (also known as the GAD-2), which are good (Adzrago, Walker, & Williams, 2024). Given literature suggesting that calculating Cronbach’s alpha for two-item measures is not feasible, we opted to determine the split-half reliability of the PHQ-2 by calculating the Spearman-Brown coefficient (Eisinga, te Grotenhuis, & Pelzer, 2013). Our results indicate an overall “good” reliability score of r_2_= 0.79. This has been included in the manuscript (pg. 33).

3.) Similarly, given that the key anxiety outcome here was only two items from the PHQ-9, some justification should be provided about why this measure was chosen as an outcome relative to other, more well-validated and robust measures of anxiety.

This justification has been provided on pg. 16 and is copied here: “This measure was originally selected for its good reliability and to minimize burden on youth participants. “

4.) It's not entirely clear how this study is "mixed methods" research, other than simply using both qualitative and quantitative methods to evaluate the app. If the authors want to refer to the study as mixed methods, they should explain what approach they took (exploratory sequential, explanatory sequential) and how the results of the quantitative results informed the qualitative work or vice versa.

Although our study technically meets criteria for a convergent parallel mixed methods design, we are hesitant to include this claim as we did not preregister it as the planned study design. We acknowledge this as an oversight. However, we had detailed our intention to use triangulation as our primary method of mixed methods integration in both our manuscript and preregistration, an approach common to convergent parallel designs (Fetters, Curry, & Creswell, 2013; Creswell & Plano Clark, 2011). Increasingly, this type of mixed methods approach is recommended for pilot intervention trials designed to examine preliminary intervention efficacy, alongside feasibility and acceptability (Aschbrenner, Kruse, Gallo, & Plano Clark, 2022). The quantitative data provide data-driven benchmarks, while the richness of the qualitative data allows us to probe participant experiences and perspectives more deeply. For example, although preliminary quantitative data in a small open trial might broadly show if anxiety changed over time, it cannot explore which features of the intervention or implementation model participants believe drove this change. Though it remains impossible to establish a causal relationship in such a trial, the qualitative data offers critical insights to contextualize quantitative findings and inform future work. This information has been added to the introduction (pg. 8).

To clarify the complementary nature of our quantitative and qualitative results, we have added a table in the Supplement (referenced in the main text on p. 9) based on previous work by Aschbrenner and colleagues (2022). This table demonstrates which data sources were used in the triangulation process for each study metric.

Supplementary Table 4.1: Triangulation of data sources

|  | Quantitative Data | Qualitative Data |
| --- | --- | --- |
| Intervention feasibility | - Protocol Adherence (Mentees)   - Percentage of enrolled mentees that complete at least ½ of assigned CBM-I training sessions   - Percentage of enrolled mentees the complete all assigned CBM-I sessions - Clinical deterioration (Mentees)   - Percentage of mentees who experienced an increase in symptoms on the anxiety subscale of the GAD-2 > 50% above their pre-intervention score | Mentee interview  “Were there any parts of the app that were hard to use?”  “How often did you use the app?” |
| Implementation framework feasibility | - Protocol Adherence (Mentors & Mentees)   - Percentage of mentor/mentee check-ins completed   - Number of times dyads discussed MindTrails during competed check-ins | Mentor Interview  “What was it like incorporating MindTrails Teen app into your mentoring sessions?”  “How often did you talk about MindTrails Teen with your mentee?”  Mentee Interview  “How often did you talk about MindTrails Teen with your mentor?” |
| Intervention acceptability | - Average responses to the following items on the MAQ: Mentee version   - “I enjoyed using the MindTrails App.”   - “I would recommend MindTrails to other mentees or teens.”   - “MindTrails changed the way I think during anxiety-provoking situations.” | Mentee Interview  “Tell us about your experience using the MindTrails Teen app. What was your (favorite/least favorite) part of the app?” |
| Implementation framework acceptability | - Average responses to the following items on the MAQ: Mentee version   - “I enjoyed working with my mentor to apply the skills I learned in MindTrails.”.”   - “I would recommend using MindTrails with a mentor to other mentees or teens.”   - “It was helpful to have my mentor support my use of MindTrails.” - Average responses to the following items on the MAQ: Mentor version   - “I enjoyed participating in this project.”   - “I felt prepared to help my mentee use MindTrails.”   - “I would recommend MindTrails with mentor assistance to other mentees.” | Mentor Interview  “What was (challenging/helpful) about incorporating the MindTrails Teen app into your mentoring sessions?”  “In what ways, if any, did participating in the MindTrails program change your relationship with your mentee?”  Mentee Interview  “Do you feel like your mentor was helpful in encouraging you to use the app? If yes, how so? If no, why?”  “How did using the MindTrails Teen with your mentor change your relationship with your mentor?” |
| Intervention outcomes | - Pre- to post-intervention change in target engagement (positive and negative interpretation bias) on the Recognition Rating Task - Pre- to post-intervention change in anxiety symptoms on the GAD-2. | Mentee Interview  “Would you recommend the MindTrails Teen app to other teenagers struggling with anxiety? Why?”  “Overall, how would you describe the MindTrails Teen app to a friend?” |

5.) Some of the conclusions overstate the strength of evidence these findings provide supporting the promise of the app. For example, the discussion says "Qualitative results corroborate the quantitative anxiolytic effect found for the app," suggesting that the data show that the app is responsible for the changes in anxiety. With most participants using the app only a few times (how long in duration, total?), why would app use be most likely to explain the changes in anxiety? Other potential explanations for this decrease in anxiety are not meaningfully discussed (other than briefly mentioned in the limitations), such as increasing the frequency of interactions with their mentor or even simply enrolling in a study on anxiety. As another example, referring to changes in anxiety as "robust" may also be an overstatement, given that the study only collected a two-item measure of anxiety at two timepoints among 10-15 participants.

We thank the reviewer for this suggestion. We have softened much of our conclusive and/or causal language throughout the Discussion. We have also expanded our discussion of the study findings to explore other explanations for reported decreases in anxiety (pg. 47).

“Besides time or regression to the mean, increasing interactions with their mentor or simply enrolling in a study on anxiety may have contributed to youth improvement on the GAD-2. It is also possible that change in the mentoring relationship during the study may have contributed to the anxiety reduction.”

6.) Finally, I realize that some of the difficulty recruiting participants may have been exclusively due to research-related procedures (e.g., the need to get consents from parents, mentors, and assent from mentees, the need for live training, etc.), but framing these results as though they support the feasibility of this approach in practice may also be a stretch, given that so few agreed to participate. The potential implications for this finding, and its implications for scaling this model outside the research context, should be meaningfully discussed as well.

We have expanded our discussion of the implications of this finding in the Discussion (pg. 49-50)

“In addition to exploring this avenue, future work could prioritize forging partnerships with mentoring organizations prior to study recruitment and working directly with key implementation stakeholders (e.g., program directors, mentors) to evaluate and improve the proposed recruitment and implementation strategy. Our difficulty recruiting mentor/mentee dyads from individual programs may suggest that top-down recruitment approaches (e.g., partnering with parent organizations) will be more fruitful than recruiting from small, local branches. It will be critical to establish reliable recruitment pathways for MindTrails Teen for it to one day serve as a feasible, accessible, and scalable intervention.

Sincerely,

Emma Wolfe, M.A.

Citations:

Adzrago D, Walker TJ, Williams F. Reliability and validity of the Patient Health Questionnaire-4 scale and its subscales of depression and anxiety among US adults based on nativity. BMC Psychiatry. 2024 Mar 18;24(1):213. doi: 10.1186/s12888-024-05665-8. PMID: 38500115; PMCID: PMC10949792.

Aschbrenner KA, Kruse G, Gallo JJ, Plano Clark VL. Applying mixed methods to pilot feasibility studies to inform intervention trials. Pilot Feasibility Stud. 2022 Sep 26;8(1):217. doi: 10.1186/s40814-022-01178-x. PMID: 36163045; PMCID: PMC9511762.

Creswell, J.W. and Plano Clark, V.L. (2011) Designing and Conducting Mixed Methods Research. 2nd Edition, Sage Publications, Los Angeles.

Eisinga R, Grotenhuis Mt, Pelzer B. The reliability of a two-item scale: Pearson, Cronbach, or Spearman-Brown? Int J Public Health. 2013 Aug;58(4):637-42. doi: 10.1007/s00038-012-0416-3. Epub 2012 Oct 23. PMID: 23089674.

Fetters MD, Curry LA, Creswell JW. Achieving integration in mixed methods designs-principles and practices. Health Serv Res. 2013 Dec;48(6 Pt 2):2134-56. doi: 10.1111/1475-6773.12117. Epub 2013 Oct 23. PMID: 24279835; PMCID: PMC4097839.

Weil LG, Fleming SM, Dumontheil I, Kilford EJ, Weil RS, Rees G, Dolan RJ, Blakemore SJ. The development of metacognitive ability in adolescence. Conscious Cogn. 2013 Mar;22(1):264-71. doi: 10.1016/j.concog.2013.01.004. Epub 2013 Jan 30. PMID: 23376348; PMCID: PMC3719211.

Wells A, Carter K. Preliminary tests of a cognitive model of generalized anxiety disorder. Behav Res Ther. 1999 Jun;37(6):585-94. doi: 10.1016/s0005-7967(98)00156-9. PMID: 10372471.

Wray-Lake L, Crouter AC, McHale SM. Developmental patterns in decision-making autonomy across middle childhood and adolescence: European American parents' perspectives. Child Dev. 2010 Mar-Apr;81(2):636-51.
