# Supplementary material for: A mixed methods evaluation of a pilot open trial of a mentor-guided digital intervention for youth anxiety
Source: PLOS Digit Health. 2026 Feb 23;5(2):e0001187. doi: 10.1371/journal.pdig.0001187 (PMC12928454; doi:10.1371/journal.pdig.0001187)
Supplement: Attachment — Submitted filename: Reponse to Reviewer December.docx [file pdig.0001187.s005.docx]

We thank the reviews for their continued careful attention to this manuscript. Please see below for our response to the proposed change from Reviewer 3:

1) “The mixed-methods integration is clearer, though one summarizing sentence in the main text could help readers quickly understand your triangulation approach.”

We thank the reviewer for identifying this area for improvement. We have added this

sentence to the main text.
